# Supplementary material for: Phytoglobin Expression Alters the Na+/K+ Balance and Antioxidant Responses in Soybean Plants Exposed to Na2SO4
Source: Int J Mol Sci. 2022 Apr 7;23(8):4072. doi: 10.3390/ijms23084072 (PMC9031766; doi:10.3390/ijms23084072)
Supplement: Supplementary file 1 [file ijms-23-04072-s001.zip › 9. Supplementary Figures (April 4).pptx]

## Slide 1
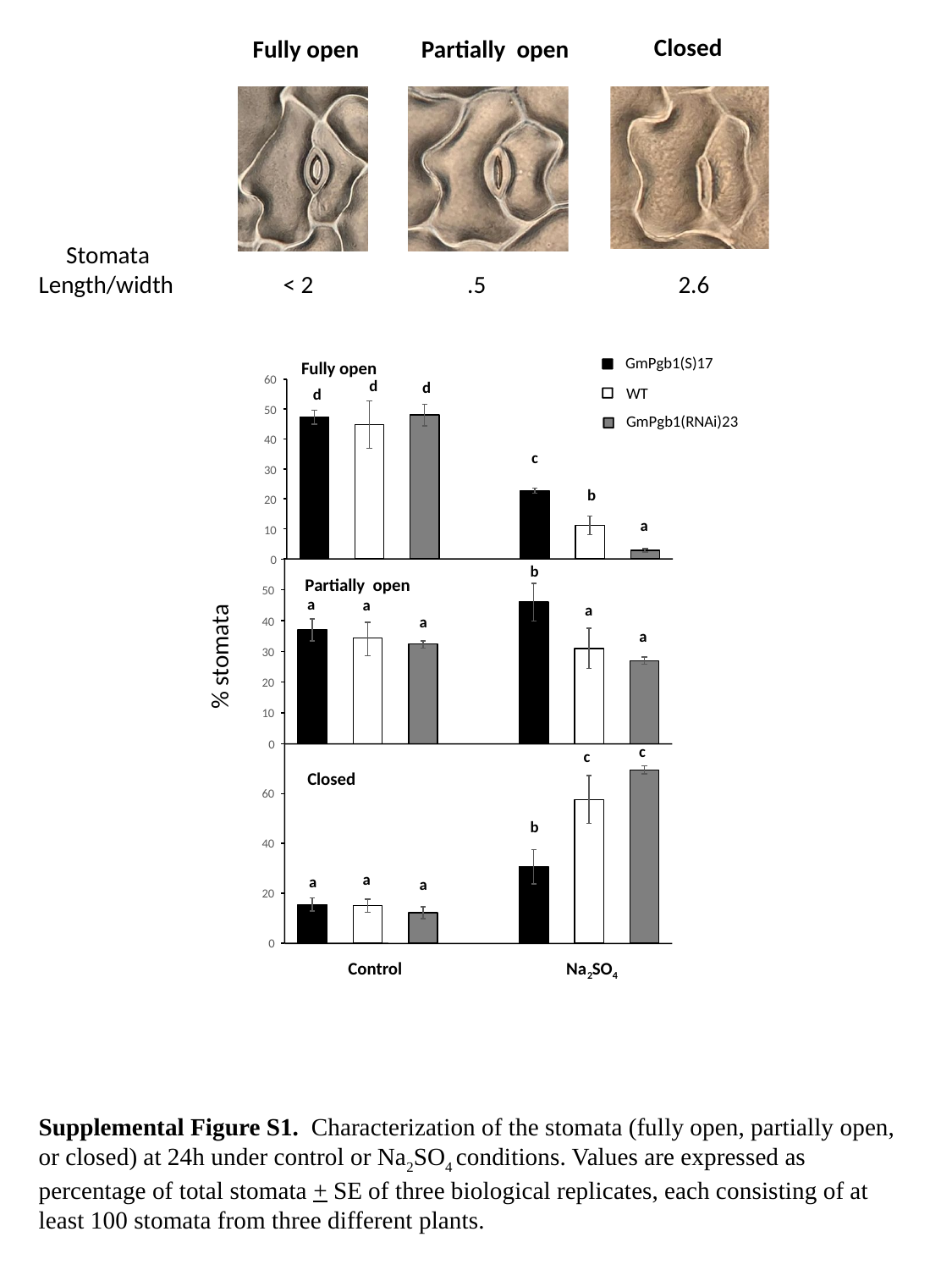

Closed
Fully open​
Partially open
 Stomata
Length/width < 2 .5 2.6
Fully open
 GmPgb1(S)17
 WT
 GmPgb1(RNAi)23
d
d
60
50
40
30
20
10
0
d
c
b
a
b
Partially open
50
a
a
a
a
40
a
% stomata
30
20
10
c
0
c
60
40
20
0
Closed
b
a
a
a
 Control Na2SO4
Supplemental Figure S1. Characterization of the stomata (fully open, partially open, or closed) at 24h under control or Na2SO4 conditions. Values are expressed as percentage of total stomata + SE of three biological replicates, each consisting of at least 100 stomata from three different plants.

## Slide 2
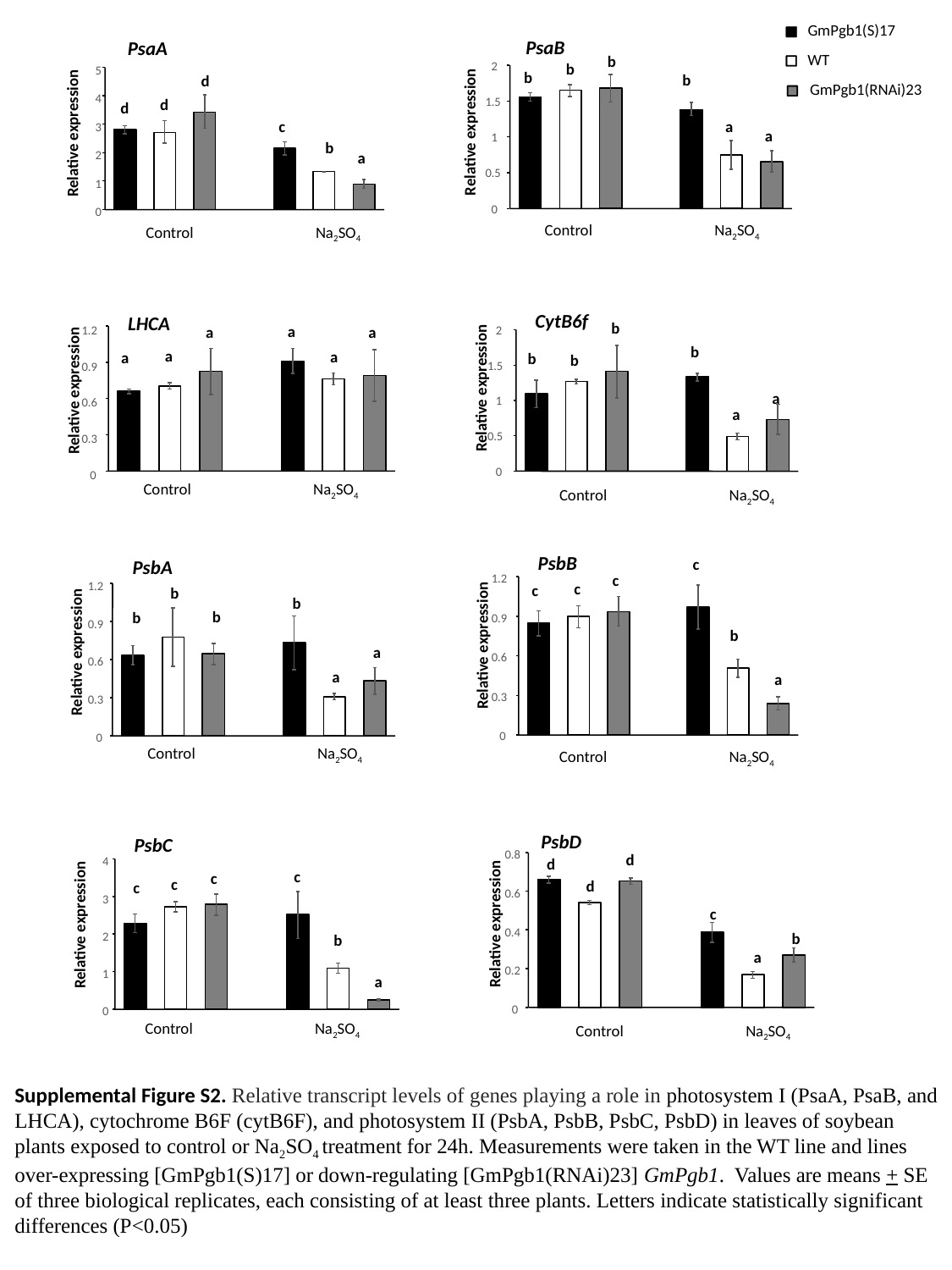

PsaB
Relative expression
 GmPgb1(S)17
 WT
 GmPgb1(RNAi)23
PsaA
b
b
2
1.5
1
0.5
0
b
5
4
3
2
1
0
b
d
d
d
c
a
Relative expression
a
b
a
 Control Na2SO4
 Control Na2SO4
CytB6f
Relative expression
LHCA
Relative expression
b
a
a
a
1.2
0.9
0.6
0.3
0
2
1.5
1
0.5
0
b
a
a
a
b
b
a
a
 Control Na2SO4
 Control Na2SO4
PsbB
Relative expression
PsbA
Relative expression
c
c
1.2
0.9
0.6
0.3
0
c
c
b
1.2
0.9
0.6
0.3
0
b
b
b
b
a
a
a
 Control Na2SO4
 Control Na2SO4
PsbD
Relative expression
PsbC
Relative expression
d
0.8
0.6
0.4
0.2
0
d
4
3
2
1
0
c
c
c
d
c
c
b
b
a
a
 Control Na2SO4
 Control Na2SO4
Supplemental Figure S2. Relative transcript levels of genes playing a role in photosystem I (PsaA, PsaB, and LHCA), cytochrome B6F (cytB6F), and photosystem II (PsbA, PsbB, PsbC, PsbD) in leaves of soybean plants exposed to control or Na2SO4 treatment for 24h. Measurements were taken in the WT line and lines over-expressing [GmPgb1(S)17] or down-regulating [GmPgb1(RNAi)23] GmPgb1. Values are means + SE of three biological replicates, each consisting of at least three plants. Letters indicate statistically significant differences (P<0.05)

## Slide 3
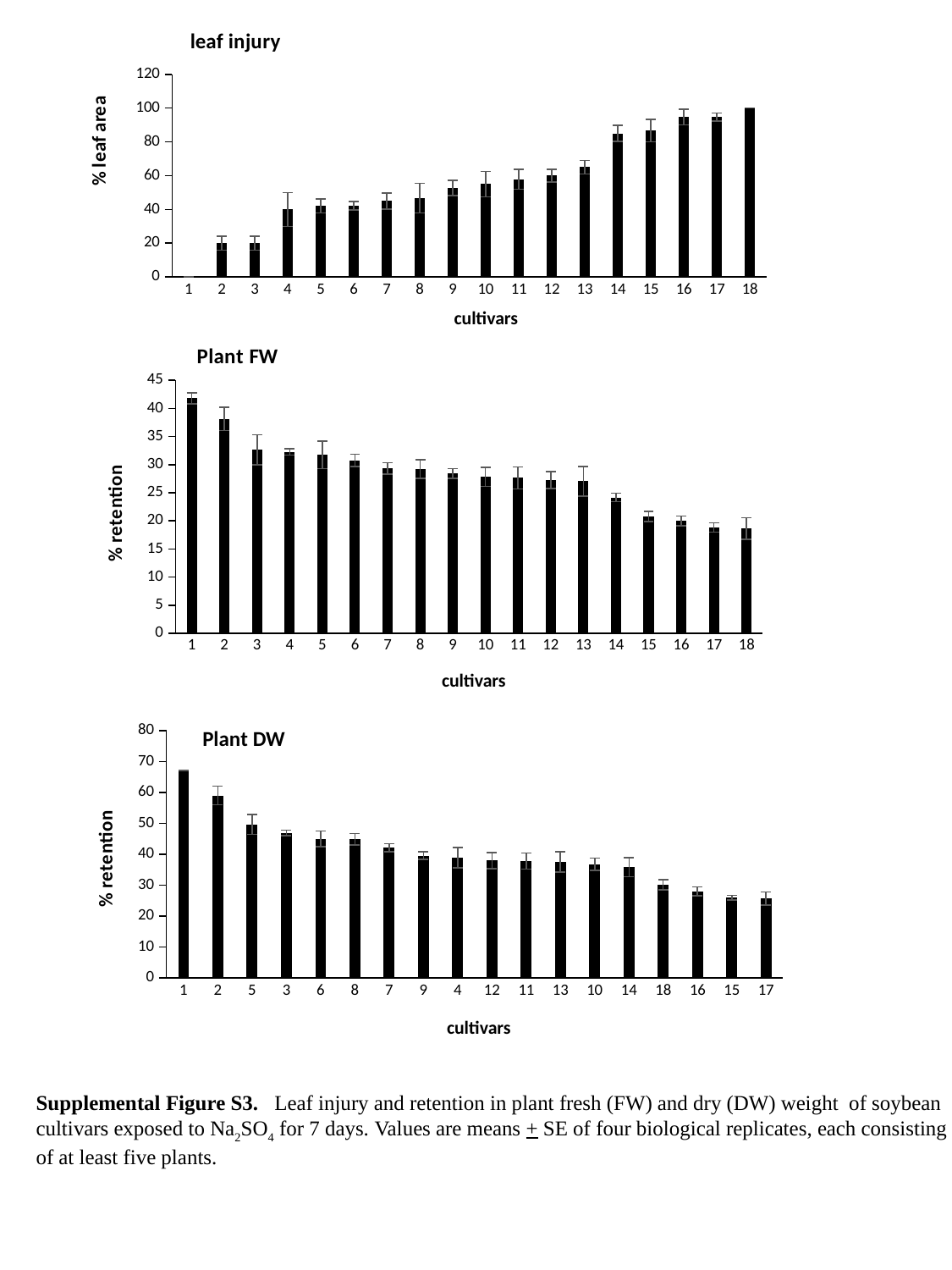

### Chart: leaf injury
| Category | injury % |
|---|---|
| 1 | 0.0 |
| 2 | 20.0 |
| 3 | 20.0 |
| 4 | 40.0 |
| 5 | 42.10526315789473 |
| 6 | 42.10526315789473 |
| 7 | 45.0 |
| 8 | 46.666666666666664 |
| 9 | 52.63157894736842 |
| 10 | 55.00000000000001 |
| 11 | 57.89473684210527 |
| 12 | 60.0 |
| 13 | 65.0 |
| 14 | 85.0 |
| 15 | 86.66666666666667 |
| 16 | 94.73684210526315 |
| 17 | 94.73684210526315 |
| 18 | 100.0 |cultivars
### Chart: Plant FW
| Category | Retention % in total fresh wt = SSI |
|---|---|
| 1 | 41.793721973094165 |
| 2 | 38.11274509803922 |
| 3 | 32.64462809917356 |
| 4 | 32.26435152374202 |
| 5 | 31.73553719008264 |
| 6 | 30.78101736972704 |
| 7 | 29.326710816777037 |
| 8 | 29.213071895424832 |
| 9 | 28.433763373190686 |
| 10 | 27.83644791666667 |
| 11 | 27.652068126520703 |
| 12 | 27.23104693140793 |
| 13 | 27.039243167484244 |
| 14 | 24.154589371980663 |
| 15 | 20.758225108225105 |
| 16 | 19.998447204968954 |
| 17 | 18.796033994334294 |
| 18 | 18.643145515183434 |cultivars
### Chart
| Category | retention % |
|---|---|
| 1 | 67.05263157894737 |
| 2 | 59.0 |
| 5 | 49.62916666666667 |
| 3 | 46.875 |
| 6 | 44.95969049373619 |
| 8 | 44.86274509803923 |
| 7 | 42.13465783664459 |
| 9 | 39.525948103792416 |
| 4 | 38.8551724137931 |
| 12 | 37.931654676258994 |
| 11 | 37.790076335877856 |
| 13 | 37.53289473684211 |
| 10 | 36.72729044834308 |
| 14 | 35.83333333333333 |
| 18 | 30.153846153846146 |
| 16 | 27.976315789473688 |
| 15 | 25.903680981595087 |
| 17 | 25.659459459459455 |Plant DW
cultivars
Supplemental Figure S3. Leaf injury and retention in plant fresh (FW) and dry (DW) weight of soybean
cultivars exposed to Na2SO4 for 7 days. Values are means + SE of four biological replicates, each consisting
of at least five plants.

## Slide 4
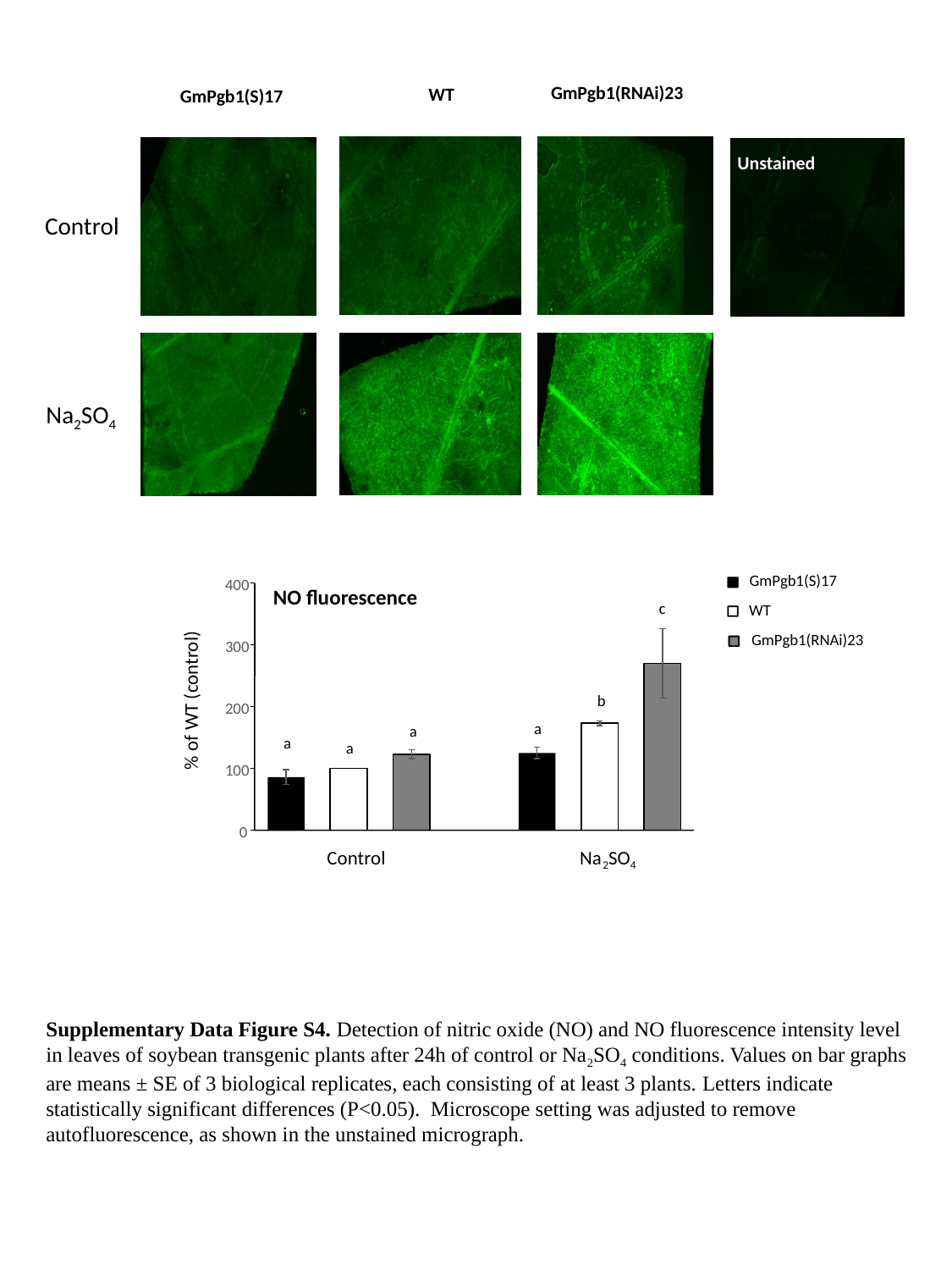

GmPgb1(RNAi)23
WT
GmPgb1(S)17
Unstained
Control
Na2SO4
 GmPgb1(S)17
 WT
 GmPgb1(RNAi)23
400
300
200
100
0
NO fluorescence
c
% of WT (control)
b
a
a
a
a
 Control Na2SO4
Supplementary Data Figure S4. Detection of nitric oxide (NO) and NO fluorescence intensity level in leaves of soybean transgenic plants after 24h of control or Na2SO4 conditions. Values on bar graphs are means ± SE of 3 biological replicates, each consisting of at least 3 plants. Letters indicate statistically significant differences (P<0.05). Microscope setting was adjusted to remove autofluorescence, as shown in the unstained micrograph.

## Slide 5
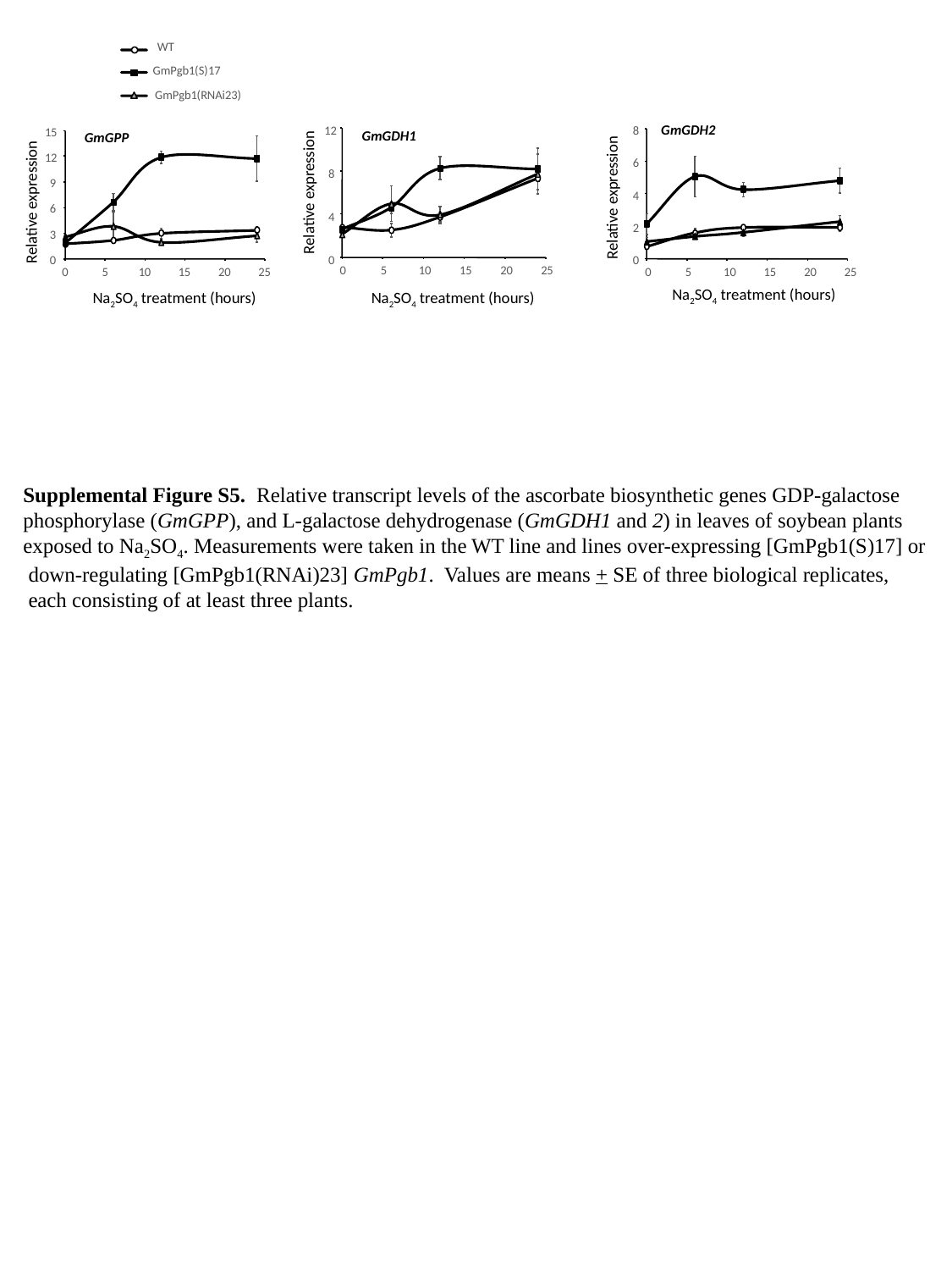

WT
GmPgb1(S)17
GmPgb1(RNAi23)
GmGDH2
Relative expression
GmGDH1
Relative expression
GmGPP
Relative expression
Na2SO4 treatment (hours)
8
6
4
2
0
5
10
15
20
25
0
12
8
4
0
15
12
9
6
3
0
0
10
15
20
25
5
0
5
10
15
20
25
Na2SO4 treatment (hours)
Na2SO4 treatment (hours)
Supplemental Figure S5. Relative transcript levels of the ascorbate biosynthetic genes GDP-galactose
phosphorylase (GmGPP), and L-galactose dehydrogenase (GmGDH1 and 2) in leaves of soybean plants
exposed to Na2SO4. Measurements were taken in the WT line and lines over-expressing [GmPgb1(S)17] or
 down-regulating [GmPgb1(RNAi)23] GmPgb1. Values are means + SE of three biological replicates,
 each consisting of at least three plants.

## Slide 6
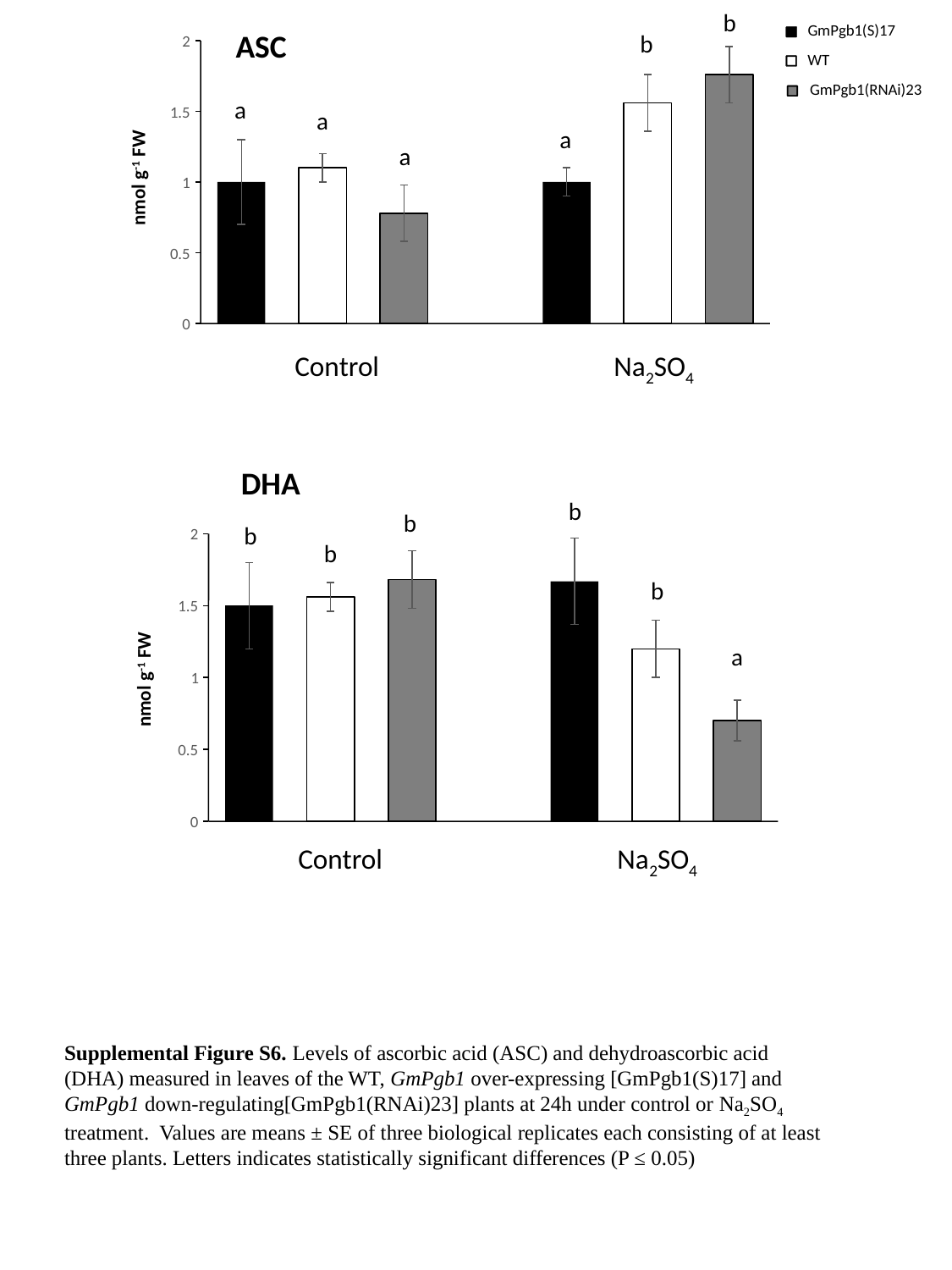

b
ASC
 GmPgb1(S)17
 WT
 GmPgb1(RNAi)23
b
2
1.5
1
0.5
0
a
a
a
a
nmol g-1 FW
 Control Na2SO4
DHA
b
b
b
2
1.5
1
0.5
0
b
b
a
nmol g-1 FW
 Control Na2SO4
Supplemental Figure S6. Levels of ascorbic acid (ASC) and dehydroascorbic acid (DHA) measured in leaves of the WT, GmPgb1 over-expressing [GmPgb1(S)17] and GmPgb1 down-regulating[GmPgb1(RNAi)23] plants at 24h under control or Na2SO4 treatment. Values are means ± SE of three biological replicates each consisting of at least three plants. Letters indicates statistically significant differences (P ≤ 0.05)
