# Supplementary material for: Phytoglobin Expression Alters the Na+/K+ Balance and Antioxidant Responses in Soybean Plants Exposed to Na2SO4
Source: Int J Mol Sci. 2022 Apr 7;23(8):4072. doi: 10.3390/ijms23084072 (PMC9031766; doi:10.3390/ijms23084072)
Supplement: Supplementary file 1 [file ijms-23-04072-s001.zip › 9. Supplementary table 1 April 4.pdf]

Table S1. Primers used for qRT-PCR analysis

Gene	Forward primer	Reverse primer
GmPgb1	AGTTCAGCTGCGGAAGGCCG	TGCTCGTTTGCTACGCCGGT
GmRBOHB	GGAAGGAGATGCTCGATCTGC	GTCTTCACCCTTGTCCCTGAAAC
GmRBOHD	CTATACGGATGCGAAAGGC	CAGTGCTAGAACATTTCCTGGG
GmRBOHG	GAACACTCGGAGACTGGAGC	GGACATCCCTGTGATCGTG
GmSOD1	ACCAATGGTTGCCTCTCAAC	CGCCCTTCCTATGATGTTGT
GmSOD3	TTCCCAAGCTTCTTCACAGG	TTCACCCCAGTGCATATCAA
GmCAT1	AAGTGTGCCCATCACAACAA	TCATGCACTTTTCACGCTTC
GmCAT2	TGCAGAAAGGTTCCCCATAC	GAGAACGGTCAGCCTGAGAC
GmAPX1	TTTGAGCTACGCCGATTTCT	TGACCCCCAGATAGAGCAAC
GmMDHAR1	TGCCCGGAGATATGAAGAAC	CCGAAACTGACCATCAACCT
GmMDHAR4	TGGTGGGAATTGGAATACG	TGCTTTGACTGGAAATGCTG
GmDHAR1	GAGATTGCTTTGGGGCATTA	TTCCACTTTAGGACGCCAAC
GmGR1	TTCCACTATCTCGCCCCTCCT	ACGGGGTCGGCGCCGTTTTG
GmGR2	TTCCTCTATCTCGTCCCTCCC	GCGGCATCGCGGCCGTTTTG
GmSOS1	CACTTGGAGCTGTAGGAATG	CCTCCTGAGCAGTGAAATAAG
GmAKT1	CCCTTTCCATAACCGATAACA	CGGTCATCCACGATCAAATA
GPP	TCTGCTGAGGAGGCTATGGT	ATCCCGATCCACCTTTCTCT
GDH1	CCGAGGAATTCGTACGTTGT	CTAAGGAGCCGAACTCGATG
GDH2	CATCGAGTTCGGCTCCTTAG	CAACCCCTTTGGTCTTCAAA
PsaA	AGCAACTCCCTTTTTCACC	GACCCGCTATCAAGAAAAGAAT
PsaB	TGGTGTTTATCAGTGGTGGT	TGATGATTGAGGCGGGATT
LHCA	ACCCATGGCACAACAACA	ACAGCACAGCGATACCAAC
CytB6F	CGTCCCTCTGTTGTCATGT	GGAGAGGTGATGGTGAAAAGTT
PsbA	GCAAACCTATAGCCGCAGA	GGATGGTTTGGTGTTTTGATGA
PsbB	CCCTCTGACCCTGTTCTT	ATATTCCAACCGCCCCAC
PsbC	CCTAGTAGTTTGCCGGAT	CACGTGGAAACGCTCTTTA
PsbD	AACGAAGTCATAGGCACG	CTTTGGGGTTGCTTTTTCC
GmHelicase	TAACCCTAGCCCCTTCGCCT	GCCTTGTCGTCTTCCTCCTCG
